# Discovery of a junctional epitope antibody that stabilizes IL-6 and gp80 protein:protein interaction and modulates its downstream signaling

**DOI:** 10.1038/srep37716

**Published:** 2017-01-30

**Authors:** Ralph Adams, Rebecca J. Burnley, Chiara R. Valenzano, Omar Qureshi, Carl Doyle, Simon Lumb, Maria del Carmen Lopez, Robert Griffin, David McMillan, Richard D. Taylor, Chris Meier, Prashant Mori, Laura M. Griffin, Ulrich Wernery, Jörg Kinne, Stephen Rapecki, Terry S. Baker, Alastair D. G. Lawson, Michael Wright, Anna Ettorre

**Affiliations:** 1 New Medicines, UCB-Celltech, 208 Bath Road, SL1 3WE, Slough UK; 2 Central Veterinary Research Laboratory, P.O.Box 597, Dubai, United Arab Emirates

## Abstract

Protein:protein interactions are fundamental in living organism homeostasis. Here we introduce VHH6, a junctional epitope antibody capable of specifically recognizing a neo-epitope when two proteins interact, albeit transiently, to form a complex. Orthogonal biophysical techniques have been used to prove the “junctional epitope” nature of VHH6, a camelid single domain antibody recognizing the IL-6–gp80 complex but not the individual components alone. X-ray crystallography, HDX-MS and SPR analysis confirmed that the CDR regions of VHH6 interact simultaneously with IL-6 and gp80, locking the two proteins together. At the cellular level, VHH6 was able to alter the response of endothelial cells to exogenous IL-6, promoting a sustained STAT3 phosphorylation signal, an accumulation of IL-6 in vesicles and an overall pro-inflammatory phenotype supported further by transcriptomic analysis. Junctional epitope antibodies, like VHH6, not only offer new opportunities in screening and structure-aided drug discovery, but could also be exploited as therapeutics to modulate complex protein:protein interactions.

The concept of the interactome and the understanding of the role played in disease by binary protein:protein interactions (PPIs) have opened alternative possibilities for therapeutic intervention^1^. Although orthosteric and in particular allosteric modulation of PPIs is considered an emerging frontier in drug discovery^2^,^3^,^4^, to date few PPIs have been found “druggable” using small molecules and peptides^5^. Due to their high specificity and affinity for a particular epitope, antibodies are a natural choice to explore the fine modulation of relevant biological axes by interfering with specific PPIs. In particular, an antibody able to stabilize a transient complex at the junction formed when the two proteins interact would be an invaluable tool for a deeper molecular understanding of PPIs, and to aid the screening or rational design of new biologicals and small molecules with improved targeting capabilities^6^.

An example of a PPI, which plays a fundamental role in the patho-physiology of several diseases, is the interaction between IL-6 and its specific receptor gp80 (also known as CD126). IL-6 binds to gp80 to form a heterodimer; this first step is characterized by fast association and dissociation phases^7^. The IL-6–gp80 complex then can bind to gp130 to form a heterotrimer, which in turn dimerizes to create the active hexameric complex responsible for key downstream signaling events^8^. IL-6 can signal in *cis* or *trans*, depending on whether gp80 is cell membrane-expressed or in a soluble form generated by shedding^9^. Both forms of gp80 are active and bind IL-6^10^. *In vitro* data suggest that the *trans*-signaling pathway is preferentially activated during inflammation^11^. Unlike gp130, which is expressed in a variety of cells, gp80 is expressed mainly on the plasma membrane of hepatocytes and immune cells^10^. Neither IL-6 nor gp80 binds to gp130, indicating that IL-6 is presented by gp80 in the appropriate conformation to then recruit gp130^10^.

IL-6 mediates an array of immune responses, including lymphocyte trafficking, proliferation and differentiation of T cells, antibody production from B cells, liver regeneration, expansion/differentiation of bone marrow progenitors, and the broad regulation of inflammatory response^12^,^13^. IL-6 is a key mediator in a variety of diseases, including cancer and autoimmunity^14^. As such, IL-6 has been targeted directly (IL-6 blocking antibodies) or indirectly (gp80 blocking antibodies and gp130-Fc) for therapeutic interventions against autoimmune disorders and cancer^15^,^16^,^17^,^18^,^19^,^20^. Using the interaction between IL-6 and gp80 as a well-known example of a PPI, we have discovered and characterized VHH6, a prototype locking antibody. VHH6 specifically recognizes an epitope that spans the junction between IL-6 and gp80, locking them together in a stable functional unit.

## Results

### VHH6 recognizes specifically the IL-6–gp80 complex

**Figure 1:**
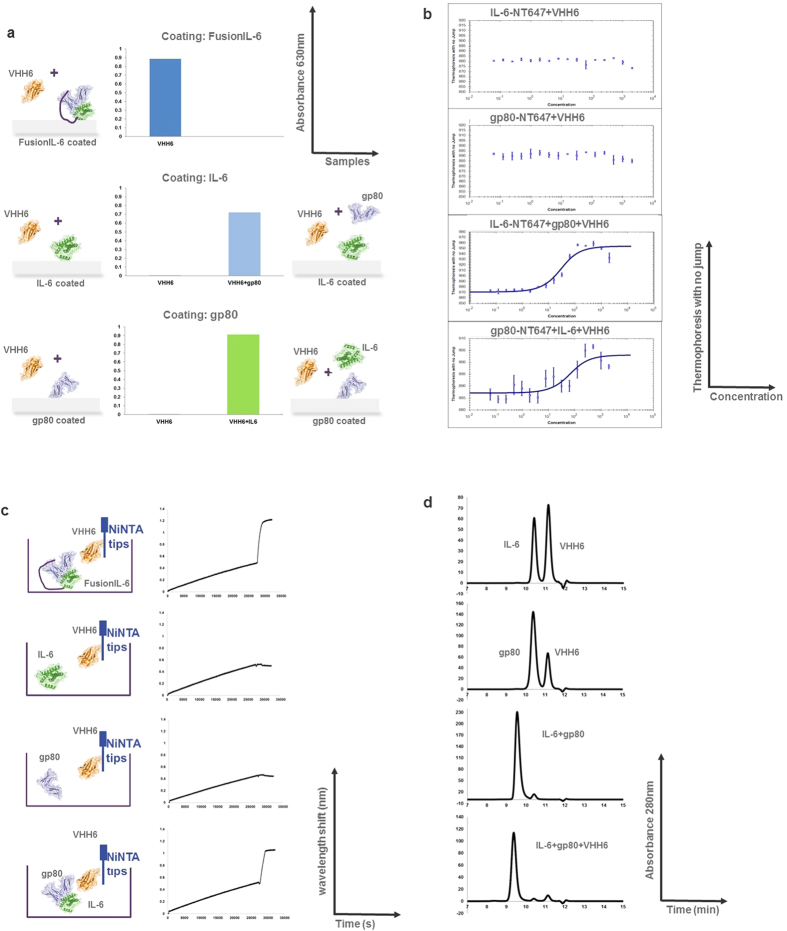
Preliminary assays to prove that VHH6 only recognizes the IL-6–gp80 complex. (**a**) **ELISA:** FusionIL-6, IL-6 or gp80 were immobilized overnight on plates. VHH6 binds to FusionIL-6, but not to IL-6 or gp80 alone. When the plates were coated with IL-6 and VHH6 was added with gp80, or when VHH6 was added with IL-6 (on gp80-coated plates), the absorbance was comparable to that of FusionIL-6. y-axis: Absorbance at 630 nm; x-axis: samples. **(b) MicroScale Thermophoresis (MST):** IL-6 and gp80 were labelled with NT647. A binding curve was apparent only when different concentrations of VHH6 were incubated with a mixture of IL-6-NT647+gp80 or gp80-NT647+IL-6 (bottom two panels), but not when either IL-6-NT647 or gp80-NT647 alone were probed. y-axis: thermophoresis intensity; x-axis: VHH6 concentration (nM). **(c) Bio-Layer Interferometry (Octet):** VHH6 was captured through its His-tag onto Ni-tips, and solutions of FusionIL-6, IL-6, gp80 and IL-6+gp80 were probed. A change in the interference pattern for VHH6 was recorded only for FusionIL-6 or IL-6+gp80 solutions. y-axis: wavelength shift (nm); x-axis: time (s). **(d) SEC isolation of VHH6 in complex with IL-6 and gp80.** Solutions of IL-6, gp80 and VHH6 alone and in combination were subjected to SEC and analyzed as described in Materials and Methods. SEC on IL-6+VHH6 and gp80+VHH6 give two separate peaks corresponding to the elution time of the single loaded components, while the elution time of VHH6–IL-6–gp80 was compatible with the elution of the trimeric complex when compared to the elution of the dimeric complex IL-6–gp80 (respectively, 9.378 min *vs.* 9.556 min). y-axis: Absorbance at 280 nm; x-axis: time (min).

Immunization of camels with HyperIL-6, a fusion protein of gp80 joined to IL-6 with a flexible peptide linker^21^, and subsequent antibody discovery led to the generation of 41 unique VHH sequences^22^. All 41 VHHs recognized HyperIL-6 and FusionIL-6 in an initial ELISA screen. FusionIL-6 is an in-house designed fusion protein between IL-6 and gp80^23^. These 41 VHHs were separated according to their ability to bind to either IL-6 (30 VHHs) or gp80 (7 VHHs)^22^. Four VHHs recognized only FusionIL-6, but neither IL-6 nor gp80 alone^22^. Of the latter, VHH6 was taken forward for further characterization, due to its superior yield in a transient expression system. Data generated using ELISA (Fig. 1a), MicroScale Thermophoresis (MST, Fig. 1b), Bio-Layer Interferometry (Octet, Fig. 1c) and analytical size exclusion chromatography (SEC, Fig. 1d) showed that VHH6 is able to recognize and bind only to the IL-6–gp80 complex.

### VHH6 increases the stability of the IL-6–gp80 complex

**Figure 2:**
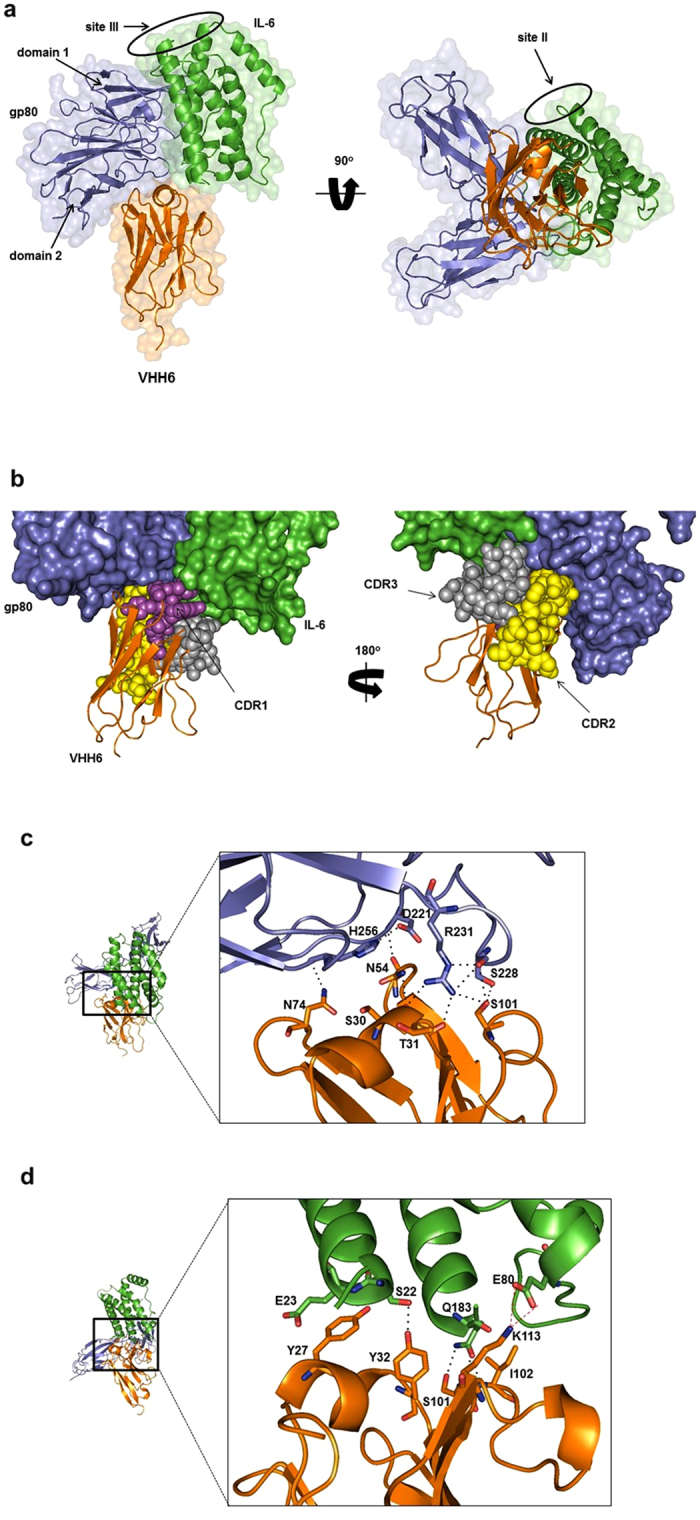
Crystal structure of the VHH6–IL-6–gp80 complex. (**a**) Crystal structure of VHH6 (orange) bound simultaneously to IL-6 (green) and gp80 (blue). The epitope covers a total surface area of 924 Å^2^, with the interface between VHH6 and IL-6 contributing 414 Å^2^, and the interface between VHH6 and gp80 contributing 510 Å^2^. Superimpositions of free IL-6 (1ALU) and free gp80 (1N26) backbone carbon alpha atoms over the equivalent atoms of VHH6–IL-6–gp80 showed no significant shifts in conformation, with root mean squared deviation (r.m.s.d.) values of 1.2 Å and 1.5 ± 0.1 Å, respectively. Right, complex rotated by 90° around x-axis. (**b**) CDR1 (magenta) and CDR3 (grey) make contacts with both IL-6 (green) and gp80 (blue), whereas CDR2 (yellow) contacts only gp80. **(c)** At the gp80 interface, CDR3 residue Ser101 and CDR2 residues Ser30 and Thr31 (both main-chain oxygen atoms) form a hydrogen bond network with Ser228 and Arg231; the main chain oxygen atom of CDR2 residue Asn54 forms a hydrogen bond with the main chain nitrogen atom of Asp221; and framework residue Asn74 forms a hydrogen bond with the main chain oxygen atom of His256. (**d**) At the interface with IL-6, CDR3 residues Ser101 and Ile102 (main-chain nitrogen atom) forms a hydrogen bond with Gln183; CDR3 residue Lys113 forms a salt bridge with Glu80; there is an aliphatic interaction between Tyr27 and Glu23; and CDR1 residue Tyr32 forms hydrogen bonds with Ser22 (see SI Fig. 1 for more details).

Four independent biophysical techniques strongly indicated that VHH6 specifically recognizes the complex between IL-6 and gp80. This suggested that VHH6 either recognized a conformational shift in one of the proteins or an epitope at the interface of the IL-6–gp80 complex. To understand the nature of the epitope, the crystal structure of the VHH6–IL-6–gp80 complex was determined (SI Table 1A). The complex crystallized in space group C 121 and was refined to 2.7 Å. The crystal structure confirmed that VHH6 binds to an epitope that spans across the interface of IL-6 and gp80. More specifically, it binds to the junction of IL-6 and domain I of gp80, with sites II and III on IL-6 remaining accessible for binding by gp130 (Fig. 2a). VHH6 binds to an epitope which is split spatially across IL-6 and gp80 almost evenly (45% of the epitope surface area lying on IL-6 and 55% on gp80).

All three complementarity-determining regions (CDRs) are involved in binding (Fig. 2b) Binding to IL-6 is mediated though multiple hydrogen bonds from the side chains of Tyr27, Tyr32 and Ser101 and the backbone NH group of Ile102 (Fig. 2c). There is also a single salt bridge from Lys113 and a likely aliphatic interaction between Tyr27 and Glu23 of IL-6. Binding to gp80 is mediated through multiple hydrogen bonds from the backbone CO groups of Ser30, Thr31 and Asn54, and the side chain of Asn74 (Fig. 2d).

To assess the contributions of the side chains to binding, single alanine substitutions at positions 27, 32, 74, 101 and 113, and double alanine substitutions at positions 27/32, 74/101 and 113/101 were generated. Either His-tagged VHH6 or mutant forms were captured from supernatant of transiently transfected Expi-HEK cells on a nickel charged NTA chip and an equimolar mixture of IL-6 and gp80 injected over a 300 s injection period. In a three-component system, it is not possible to calculate meaningful association rate constants. However, the dissociation rate constants, being concentration independent, were determined. For single residue mutants, the dissociation rates were very similar to that of VHH6, except for the Y27A mutant, which showed a 1.9-fold increase. The dissociation rates for the double mutants all increased, with 3.8- and 4.3-fold increases observed for the double mutants Asn74Ala/Ser101Ala and Ser101Ala/Lys113Ala respectively (SI Table 1B,C). These data confirmed the role in binding of the contacts identified in the crystal structure.

**Figure 3:**
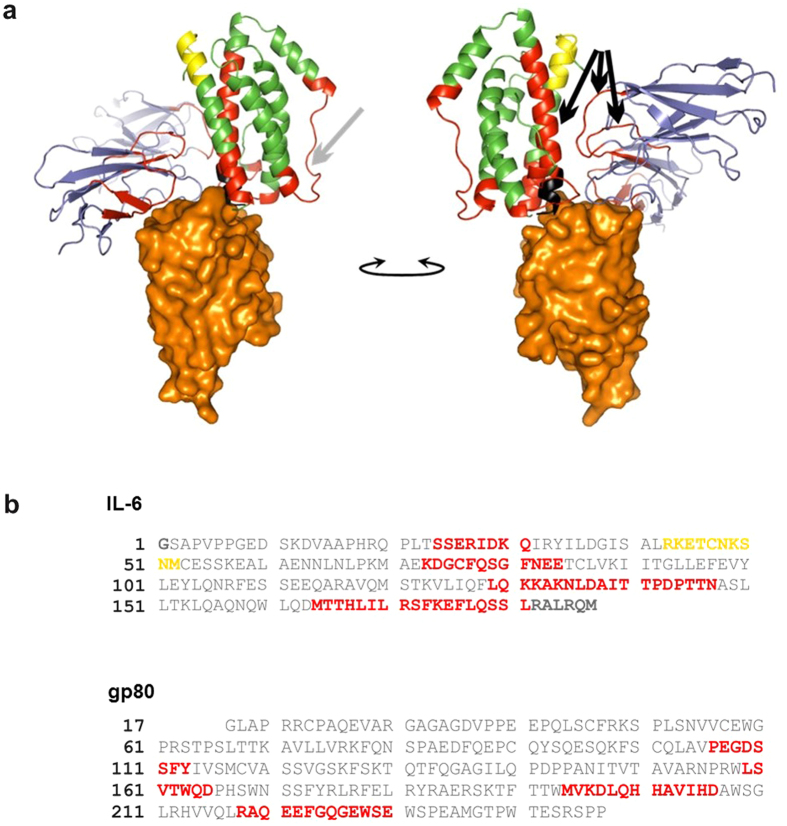
HDX-MS of the VHH6–IL-6–gp80 complex confirms the junctional epitope identified by crystallography and indicated reduced structural dynamics at the IL-6–gp80 interface. HDX-MS was applied to map the epitope of the VHH by comparing the HDX for a purified IL-6–gp80 complex with that for a purified VHH6–IL-6–gp80 complex. After the exchange reaction, the proteins were digested by pepsin and the resulting peptides analyzed by LC/MS. Sequence coverage of 99.6% was achieved for gp80 and 94.1% for IL-6. No data were obtained for the residues 179–184 (RALRQM) at the C-terminus of IL-6, also expected to interact directly with the VHH. HDX-MS data are represented on **(a)** the crystal structure of VHH6–IL-6–gp80 and (**b**) the sequences of IL-6 and gp80. Peptides with differential-deuterium uptake in the presence and absence of the VHH6 with p < 0.01 (Student’s t-test) for ≥2 time-points are defined as significant. Regions showing a decrease in HDX-MS in the presence of VHH6 are shown in red; the region showing an increase in HDX is shown in yellow; the region for which no HDX-MS data were obtained is shown in black. (**a**) IL-6 is shown in green, gp80 in blue and VHH6 in orange (surface representation). Left hand image: arrow indicates loop rigidified upon VHH binding; right hand image: arrows indicate regions involved in the gp80–IL-6 interface.

To understand how VHH6 affects the structural dynamics of the IL-6–gp80 interaction, we used HDX-MS^24^ (see Fig. 3a,b). A lower amount of deuterium exchange is indicative of decreased structural dynamics and/or protection of that region by a binding event, whereas higher exchange indicates an increase in dynamics. Data are obtained at the peptide level, whereby changes observed can be attributed to one or more of the residues contained therein. Lower deuterium uptake was seen in the presence of VHH6 for the sequences SSERIDKQ and KDGCFQSCFNEE (residues 21–28 and 70–81 respectively) in IL-6 and LSVTWQD and MVKDLQHHAVIHD (residues 215–221 and 250–262 respectively) in gp80, each of which contains residues located within the VHH6 interface, reinforcing that the epitope spans both proteins. No data were obtained for the residues 179–184 (RALRQM) at the C-terminus of IL6, also expected to interact directly with the VHH. Peptides in the regions MTTHLILRSFKEFLQSSL (residues 161–178, IL-6) and PEGDSSFY and RAQEEFGQGEWSE (residues 162–169 and 274–286 respectively, gp80) also showed a decrease in HDX in the presence of the VHH (Fig. 3a, right); these are located at the IL6-gp80 interface, suggesting a tightening of the interaction here resulting in reduced structural dynamics. Finally, residues 126–144, LQKKAKNLAITTPDPTTN, in IL6 are shown to be less dynamic in the presence of the VHH6 (Fig. 3a, left); indeed, these are resolved in the crystal structure of the trimer, whereas they are too flexible to be modelled in the structure of IL6 alone^25^ (1ALU), indicating once more an increase in structural rigidity here upon VHH binding. There is also an indication of an allosteric conformational/dynamical change occurring on the distal side of IL6 (RKETCNKSNM, residues 40–49). For a more complete list of overlapping peptides defined by comparing HDX patterns see SI Table 2A and B.

**Figure 4:**
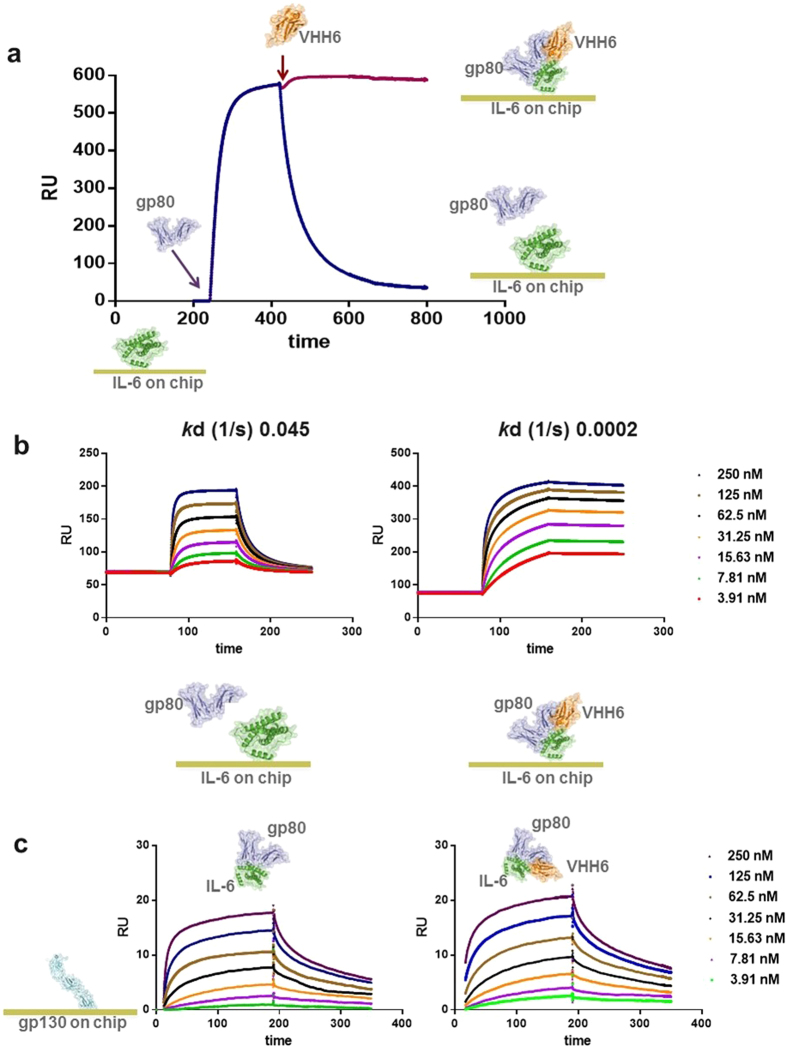
Binding of VHH6 to the IL-6–gp80 complex changes the dissociation rate of the complex but does not affect the binding of the IL-6–gp80 complex to gp130. (**a**) A qualitative SPR experiment was performed immobilizing IL-6 on a CM5 chip and injecting gp80. The blue curve represents a typical sensorgram of gp80 binding to IL-6; t red curve is the sensorgram resulting from gp80 binding to IL-6 with VHH6 injected at the beginning of the dissociation phase for gp80, showing an inhibition of gp80 dissociation curve (y-axis: RU; x-axis: time (s)). (**b**) A quantitative SPR was performed immobilizing IL-6 on a CM5 chip and injecting gp80. Different concentrations of gp80 were tested in the absence or in the presence of an excess of VHH6 (see Materials and Methods). *k*d are reported above each sensorgram (y-axis: RU; x-axis: time (s)). **(c)** gp130 was immobilized on a CM5 chip. A fixed excess concentration of IL-6 and differing concentrations of gp80 in the presence or absence of VHH6 were tested. IL-6–gp80 complex binds gp130 with *k*a of 3.6 × 10^5^ M^−1^ s^−1^ and *k*d 0.037 s^−1^, while the VHH6–IL-6–gp80 complex binds gp130 with *k*a of 4.7 × 10^5^ M^−1^ s^−1^ and *k*d 0.029 s^−1^ (y-axis: RU; x-axis: time (s)). The data confirmed that are no significant differences either in the association or the dissociation phase of IL-6–gp80 complex binding to gp130 upon addition of VHH6.

To investigate the effect of VHH6 on the binding kinetics of the IL-6–gp80 complex, we performed a qualitative SPR experiment, immobilizing IL-6 on a CM5 chip and injecting gp80. As expected for a cytokine-receptor transient interaction, both fast association and dissociation phases were observed (Fig. 4a). Injection of VHH6 at the beginning of the dissociation phase of gp80 abrogated dissociation of gp80 from immobilized IL-6. VHH6 reduced the dissociation rate constant (*k*_d_) for gp80 binding to IL-6 by 225-fold from 0.045 s^−1^ to 0.0002 s^−1^ (Fig. 4a). For brevity, Fig. 4a and b only report the SPR sensorgrams for IL-6 immobilization. Similar results were obtained when gp80 or VHH6 were immobilized (SI Table 4). These data demonstrate that not only does VHH6 bind to the IL-6–gp80 complex, but that it significantly impedes the dissociation of IL-6 from gp80.

The model created by superimposing the crystal structure solved for VHH6–IL-6–gp80 on the published data for the complex IL-6–gp80–gp130^8^ (PDB 1P9M) indicated that VHH6 would not block the binding of IL-6–gp80 to gp130 (SI Fig. 2). To confirm this, gp130 extracellular domain was immobilized on the chip and a mixture containing IL-6–gp80 or VHH6–IL-6–gp80 was tested. As predicted, VHH6 did not impair the binding of the IL-6–gp80 complex to gp130 (Fig. 4c). Furthermore, stabilization by VHH6 did not affect the kinetics of the IL-6–gp80 complex binding to gp130.

### VHH6 modulates IL-6 signaling in a cell model for *trans*-signaling

Our next step in the characterization of VHH6 was to explore the cellular outcomes following the stabilization of the IL-6–gp80 complex. Recruitment of gp130 by IL-6–gp80 leads to a series of phosphorylation events ultimately culminating in the phosphorylation of Signal Transducer and Activator of Transcription 3 (pSTAT3) molecules. Phosphorylated STAT3 molecules are translocated to the nuclei^26^, where they activate the transcription of genes involved in the response to IL-6. IL-6-induced STAT3 phosphorylation peaks at 30 min after stimulation, and whilst largely exhausted within 60–90 min, a second wave is observed after 4 h^27^. We postulated that adding VHH6 to the IL-6 and gp80 mixture would increase the magnitude of STAT3 phosphorylation, thus mimicking the effects of HyperIL-6 *in vitro*^28^.

**Figure 5:**
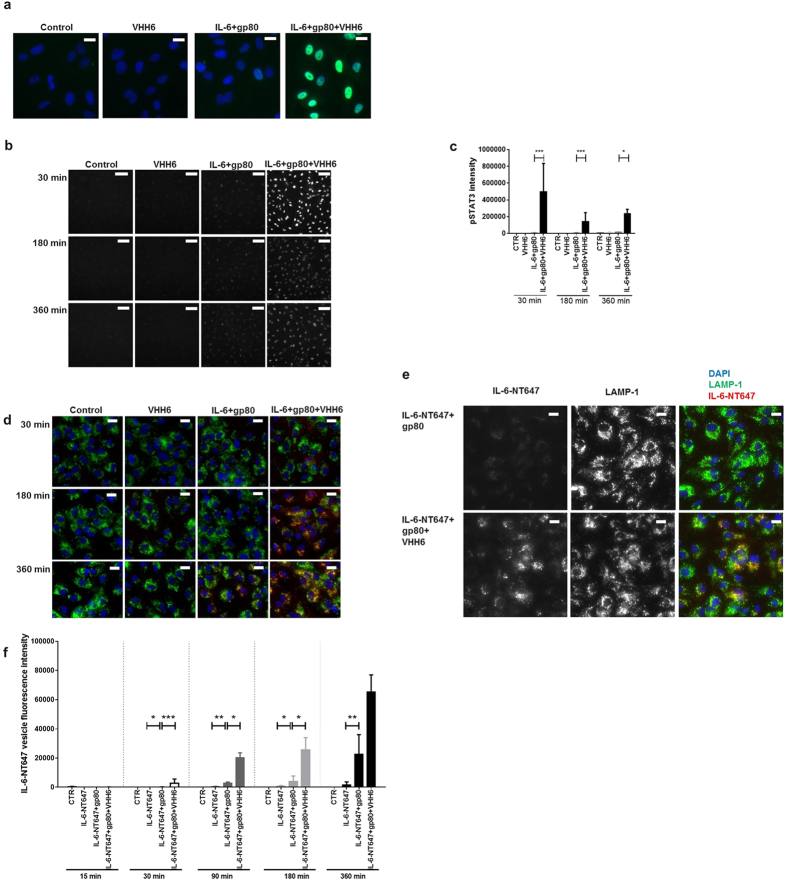
Stabilization of the complex IL-6–gp80 by VHH6 increases the internalization of IL-6-NT647 over time and promotes an increased and sustained STAT3 phosphorylation signal in HUVECs. HUVECs were treated with IL-6+gp80 and VHH6+IL-6+gp80 or IL-6-NT647+gp80 and VHH6+IL-6-NT647+gp80 as described in Materials and Methods. Both pSTAT3 and IL-6-NT647 fluorescent signal were quantified at different time points using the “spot total intensity per object” parameter. Three different replicates were performed for each time point (statistical significance: *=p ≤ 0.05; **=p ≤ 0.01 and ***=p ≤ 0.001). **(a)** A representative experiment showing pSTAT3 signal and nuclear translocation at 30 min. (Scale bar = 20 μm; Blue: DAPI, Green: pSTAT3). (**b**) A representative time-course experiment showing pSTAT3 signal at 30, 180 and 360 min. (Scale bar = 100 μm). **(c)** Time-course analysis of pSTAT3 signal over time showing that VHH6 promotes an increased and sustained pSTAT3 signal. y-axis: pSTAT3 fluorescence intensity; x-axis: time (min). IL-6 labelled with NT647 was used to track over-time internalization of the IL-6–gp80 complex. VHH6 significantly increased the rate of IL-6-NT647 accumulation in vesicles in a time-dependent manner. (**d**) A representative time-course experiment showing IL-6NT647 signal at 30, 180 and 360 min. (Scale bar = 20 μm; Blue: DAPI; Green: LAMP-1; Red: IL-6-NT647). **(e)** The accumulation of IL-6-NT647 co-localized with LAMP-1 (t = 360 min; Scale bar = 20 μm; Blue: DAPI; Green: LAMP-1; Red: IL-6-NT647). (**f**) Statistical analysis of IL-6-NT647 vesicle intensity at 15, 30, 90, 180 and 360 min. y-axis: IL-6-NT647 vesicle intensity; x-axis: treatments grouped per time point.

To have a controlled system, IL-6 and gp80 were added in a stoichiometric ratio of 1:1 to Human Umbilical Vein Endothelial Cells (HUVECs), a well-established *in vitro* system for IL-6 *trans*-signaling^29^. HUVECs were treated with IL-6+gp80 and VHH6+IL-6+gp80, and STAT3 phosphorylation was quantified at different time points using high-throughput fluorescence microscopy. FusionIL-6 was also included as an internal control in all experiments (SI Fig. 3). As expected, VHH6+IL-6+gp80 showed a greater response than the mixture of IL-6 and gp80, with comparable intensity to that of FusionIL-6 at 30 min (SI Fig. 3) and sustained effects at later time points (Fig. 5a–c). VHH6 addition to the IL-6 and gp80 mixture clearly showed an increased activation of pSTAT3 at 30 min, with pSTAT3 signal predominantly detected in the nuclear compartment (Fig. 5a). Interestingly, treatment with VHH6 promoted a significant and sustained activation of pSTAT3 at later time points (Fig. 5b,c).

STAT3 phosphorylation is an indirect way of demonstrating that the stabilization of the complex is instrumental in maximizing the effects of IL-6. Indeed, another way would be to monitor the intracellular trafficking of IL-6 in the presence or absence of VHH6. We investigated whether increased and sustained activation of STAT3 due to VHH6 was ascribable to an altered internalization of IL-6–gp80 using IL-6 labelled with NT647. Figure 5d shows a representative time-course experiment with accumulation of IL-6-NT647 in vesicles at 30, 180 and 360 min. Also, the accumulation of IL-6-NT647 co-localized with LAMP-1, indicating that IL-6NT647 has been taken up in vesicles (Fig. 5e). VHH6 promoted a significant increase in intracellular accumulation of IL-6-NT647 as early as 30 min and increasing over time when compared to that of IL-6–gp80 (Fig. 5f).

**Figure 6:**
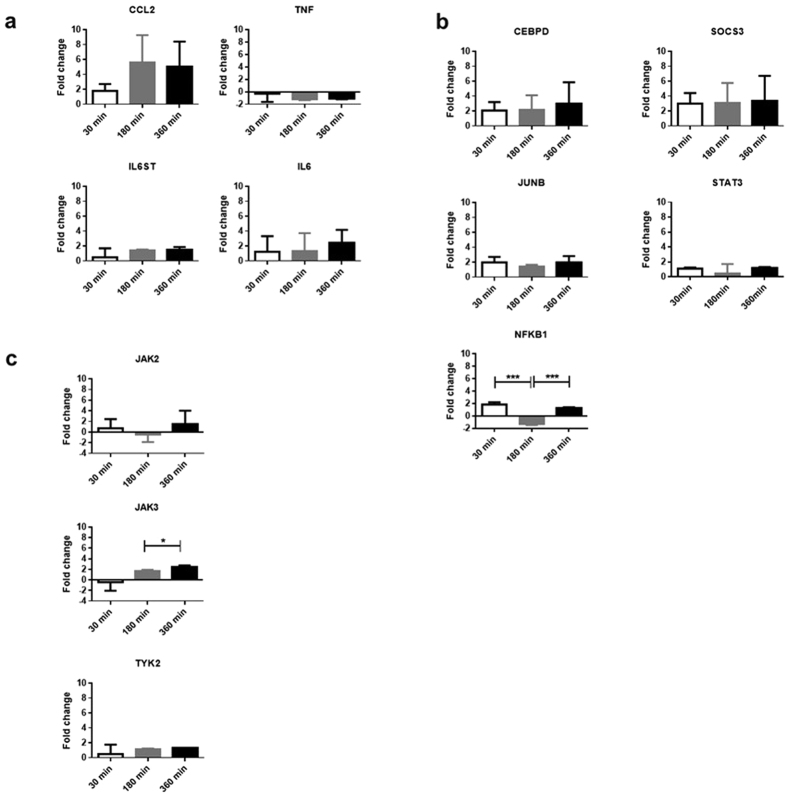
Transcriptomic analysis of HUVECs treated with VHH6–IL-6–gp80 confirms selective up-regulation of pro-inflammatory genes. Three different batches of HUVECs were analyzed at 30, 180 and 360 min. Cells treated with VHH6+IL-6+gp80 (sample) were compared to cells treated with IL-6+gp80 (control). For genes that showed either down–regulation or up-regulation, the fold changes from each batch of HUVECs were plotted. The different genes at three different time points were clustered according to cellular function: **(a)** cytokines, chemokines and chemokine receptors; **(b)** transcription factors and cytokine regulators; **(c)** kinases. y-axis: fold change; x-axis: time (min). Statistical significance: *=p ≤ 0.05; **=p ≤ 0.01 and ***=p ≤ 0.001.

To further investigate the cellular effects of extended STAT3 phosphorylation and accumulation of IL-6 in intracellular vesicles, we performed a transcriptomic profile of HUVECs using a qPCR array specifically designed for the IL-6 pathway. We compared the trans-signaling response to IL-6–gp80 treatment in the absence (control group) and presence of VHH6 (treated samples). A heat-map generated from three replicates together clearly suggested that, despite the variability intrinsically related to primary cells, VHH6 treatment affected only a few genes related to cytokine/chemokines or transcription factors in IL-6 signaling (SI Fig. 4) and is related to the length of treatment. Those genes were grouped into three categories: chemokines/cytokines, transcription factors and signaling molecules (Fig. 6a–c). Of the chemokine and cytokine genes, *CCL2, IL6* and *IL6ST* transcripts increased, whilst *TNF* decreased over time. Transcription factors genes for *CEBPD* and *JUNB* were up-regulated at all times, whilst *NFKB1* followed an up-down-up pattern. The signaling molecule *SOCS3* was up-regulated at all times; *STAT3* and *JAK2* showed a wave-like distribution, whilst *JAK3* and *TYK2* increased over time. These results pointed towards an increased inflammatory response in HUVECs due to VHH6 treatment.

## Discussion

In this study we introduce VHH6, a prototype locking antibody which recognizes an epitope newly formed at the junction where IL-6 and gp80 interact to form a transient complex^7^ before recruiting the signaling receptor gp130. This paper blends several approaches to prove the junctional epitope nature of VHH6 and its ability to stabilize the complex between IL-6 and gp80 by locking together the two proteins.

HDX-MS revealed how VHH6 alters the dynamic or “breathability”, which characterizes the structure of the IL-6–gp80 complex. X-ray crystallography confirmed that VHH6 clamps the two molecules together without interfering with the recruitment of gp130 into the signaling complex. Once the VHH6–IL-6–gp80 ternary complex is formed, IL-6 and gp80 are no longer able to dissociate, as shown by SPR experiments.

The alteration of the kinetics between IL-6 and gp80 has allowed us to solve the structure of the IL-6–gp80 complex in the absence of gp130. VHH6 engages simultaneously two proteins using its CDRs. VHH6 differs from bispecific antibodies, specifically engineered to target two proteins simultaneously. In the literature there are several examples of junctional antibodies. Diskin *et al*.^30^, described a bimolecular epitope scFv where one CDR of the VH domain contacts CD4 and one CDR of the VL domain contacts gp120. The crystal structure of a neutralizing antibody able to stabilize hemagglutinin stem was recently described by Impagliazzo *et al*.^31^, while an example of an antibody (KZ52) that engages three discontinuous segments of Ebola virus GP1 and GP2 proteins was published by Lee *et al*.^32^.

However, to the best of our knowledge, VHH6 is the prototype of an antibody which stabilizes the transient PPI between IL-6 and gp80. This is a perfect example of transient PPIs, characterized by fast association and dissociation rates, which has an important consequence from an immunological point of view to control the downstream signaling of e.g. pro-inflammatory cytokines. The key feature is that, as VHH6 binds to the complex IL-6–gp80, it effectively acts as an agonistic antibody for IL-6, as proved by our in-depth characterization, ranging from crystal structure to binding properties and to biological activity.

VHH6 works similarly to an entropic trap, making collisions between IL-6 and gp80 efficacious while increasing the concentration at equilibrium of the IL-6–gp80 complex. Our hypothesis is that VHH6, by overcoming the entropic cost required by the system to keep IL-6 and gp80 together, allows the stable complex formed to recruit gp130 and form the hexameric signaling complex.

The ability of VHH6 to alter the interaction between IL-6 and gp80 at the junction of the two molecules, without interfering with the recruitment of the signaling partner gp130, made it possible to reproduce *in vitro* some of the features of chronic inflammation using HUVECs.

In the HUVEC cell model for IL-6 *trans*-signaling, the addition of VHH6 to IL-6 and gp80 simply translates into a faster uptake and greater intracellular accumulation of IL-6 in lysosomal vesicles. Not only did VHH6 significantly increase the pSTAT3 signal within 30 min of treatment, but the effect extended to 360 min at a significantly greater level than that achieved with the mixture of just IL-6 and gp80.

Sustained STAT3 phosphorylation is associated with auto-inflammatory diseases^33^, such as rheumatoid arthritis (RA). To confirm that stabilization of IL-6–gp80 leads *in vitro* to a pro-inflammatory response, the transcriptomic profile of HUVECs treated with VHH6–IL-6–gp80 was compared to that of cells treated with IL-6–gp80. The up-regulation of genes involved in the pro-inflammatory response was directly related to the length of treatment with VHH6–IL-6–gp80. Further supporting our findings that pSTAT3 was readily translocated into the nuclei, the up-regulated genes were linked to transcription factors and cytokine activity regulators (*NFKB1, CEBPD, JUNB, STAT3 and SOCS3*), kinases of the JAK family, cytokine and cytokine receptors (*IL-6* and *IL-6ST*, alias gp130) and chemokines (*CCL2*, alias monocyte chemotactic protein 1 or MCP1). For the relevance to disease and disease biomarkers, the most interesting of these genes is the one coding for MCP1. MCP1 is up-regulated in synovia^34^ and in serum samples^35^ of RA patients. It has been shown that pro-inflammatory stimulation of HUVECs leads to the production of IL-6 and CCL2, with both contributing to an increase in endothelial permeability^36^,^37^. Therefore, the manipulation of STAT3 signaling using a junctional epitope antibody appears to reflect the modulation that can occur in a disease state.

VHH6 offers great potential in future studies. Due to its ability to stabilize and modulate the IL-6 and gp80 interaction, VHH6 could find an application in understanding the role played by IL-6 cross-talk with other cytokines or to verify the hypothesis that the switch between IL-6 (pro-inflammatory cytokine) and anti-inflammatory cytokine (IL-10), which share STAT3 as a transcription factor, might be due to an extended pSTAT3 signal^38^. It would be equally interesting to explore the therapeutic applications of VHH6 or a fusion protein VHH6-gp80 in biological systems where IL-6 plays a role as a growth factor^12^, e.g. after injury in liver regeneration^39^ or activation of hepatocyte-like progenitor cells^40^ and in expansion of hematopoietic progenitor cells^21^. Lastly, and particularly pertinent for drug discovery purposes, VHH6, by locking IL-6 and gp80 together, could be instrumental for screening more specific inhibitors (peptides, small molecules or even antibodies) of the IL-6–gp80 complex, thus leading to a selective inhibition of the IL-6 *trans*-signaling pathway in pathologies.

In conclusion, the data generated in this proof-of-concept study demonstrate the functional consequences of manipulating the interface between two proteins by stabilizing their interaction with a complex-specific antibody. Using antibodies, instead of fusion protein constructs, offers the advantage of proteins with well-defined chemico-physical characteristics and the capability of preserving the correct conformation of the complex as a functional unit. Junctional epitope antibodies like VHH6, which lock together and stabilize a transient PPI, represent excellent tools to explore novel biological hypotheses. Ultimately this may lead to a different way of approaching drug discovery and therapeutic interventions, when the target is not a single molecule but a multiprotein complex.

## Materials and Methods

*Protein production and purification*. With the exception of Human HyperIL-6^21^, all proteins (FusionIL-6, gp80V4, gp80-hscFc, IL-6 and IL-6S21) were designed in house. FusionIL-6 is a modified version of HyperIL-6 where all non-natural linker residues have been removed, and IL6 (V30-M212) was fused directly onto the C-terminus of gp80 at the end of domain 3 (S320). In addition, two unpaired cysteines were mutated to alanine (C211A and C277A). To increase the success in crystallization, an engineered form of gp80 was used (gp80V4). All those proteins were produced in house. For constructs, vectors and cell systems used for protein production see SI Table 4. Proteins used for crystallography and HDX-MS were produced in Expi-HEK or CHO cells (Gibco, Thermo Fisher Scientific) in media containing Kifunensine (GlycoSyn). IL-6 was produced in *E. coli*. For *in vitro* experiments IL-6 and gp80 were purchased from R&D Systems. For SPR experiments, gp130 was purchased from R&D Systems.

*Ni purification*. Clarified supernatant from Expi-HEK or CHO cells was passed through a 0.22 μm filter and loaded onto a 1 ml/5 ml Ni Excel column (GE Healthcare) pre-equilibrated in PBS (pH 7.4) containing 20 mM imidazole. Bound protein was eluted with PBS (pH 7.4) containing 250 mM imidazole. Fractions containing the protein of interest, as judged by 4–12% Bis/Tris NuPage (Life Technologies, Gibco, Thermo Fisher Scientific) gel electrophoresis, were dialyzed to remove imidazole and treated with TEV protease at a ratio of 1 mg per 100 mg protein. After overnight incubation at 4 °C, the sample was passed over a Ni Excel column for a second time removaling of both the non-cleaved protein and the His tagged TEV protease resulting in pure cleaved protein in the column flow through.

*Fc-tagged protein purification*. Clarified supernatant from Expi-HEK or CHO cells was passed through a 0.22 μm filter and loaded onto a 1 ml/5 ml Mab Selectsure column (GE Healthcare) pre-equilibrated in PBS (pH 7.4). If the protein was mouse Fc-tagged, 0.5 M NaCl was added to the supernatant to facilitate binding. Protein was then eluted with a phosphate/citrate pH gradient using 0.15 M Na_2_HPO_4_ at pH 9 and 0.1 mM Sodium Citrate at pH 2. Proteins were eluted at around pH 3.5 and immediately neutralized using 2 M Tris at pH 8.

TEV-HuFc tagged proteins were eluted by adding TEV protease directly to the Mab Selectsure beads at 100 μg TEV per ml of beads and incubating in PBS at 4 °C overnight. Beads were then removed by centrifugation to leave cleaved untagged protein.

*Gel Filtration*. Proteins were concentrated using Centricon Tubes (Merck Millipore) and loaded onto an S75 26/60 gel filtration column (GE Healthcare) pre-equilibrated in PBS. Peak fractions were pooled and concentrated using Centricon Tubes (Merck Millipore).

*Antibody discovery campaign*. Two camels were immunized subcutaneously over a period of three months with HyperIL-6^21^, escalating the dose from 3 μg up to a maximum of 1 mg in half log increments, at the Central Veterinary Research Laboratory (CVRL) in Dubai, according to a humane protocol approved by the Scientific Directorate of the CVRL. Experimental procedures were performed in accordance with relevant guidelines/regulations and approved by the Scientific Directorate of the CVRL. Initial immunization was carried out with complete Freund’s adjuvants, while the subsequent boosts used incomplete Freund’s adjuvants. 3 mL serum samples were collected at 8, 10, 12 and 18-week post-initial immunization to monitor the immune response; at 19-week post-initial immunization, 200 mL of heparinized blood were collected, and PBMCs were isolated and frozen. Single domain VHHs were generated from frozen PBMCs from immunized animals by cloning the variable region genes from isolated B cells using the UCB proprietary antibody discovery platform^41^,^42^. VHHs were subsequently cloned in the pIMMs vector with TEV-6His tag downstream the VHH fragment for mammalian expression^22^.

*Preliminary screening assays*. ELISA. IL-6, gp80 and FusionIL-6, used in ELISA to coat plates, were diluted to a final concentration of 2 μg/mL in carbonate buffer (0.16% Na_2_CO_3_ and 0.3% NaHCO_3_ in H_2_O). Coated plates were incubated at 4 °C overnight. The following day, the plates were washed twice in PBST, blocked in PBS/5%BSA for 2 h at RT, and again washed twice in PBST. For preliminary screening, either supernatant from HEK cells or purified VHHs was added. Anti-llama-HRP or anti-His-HRP antibody was added for 1 h at RT. Afterwards, the plates were washed twice with PBST and developed with TMB substrate. The reaction was stopped by adding NaF. Plates were read at 650 nm with a reference wavelength of 450 nm (Biotek PowerWave HT Microplate Spectrophotometer). In other experiments, IL-6 was coated overnight on the plate; VHH6 was added alone or with gp80-hscFc. Alternatively, gp80-hscFc was coated overnight on the plate, VHH6 was added alone or with IL-6. Anti-llama-HRP antibodies (Bethyl Laboratories), anti-human Fc-HRP (Jackson Lab, USA) or anti-His-HRP (Qiagen) antibodies were used for revealing the plate.

HPLC. Solutions of VHH6, gp80 and IL-6 alone or in combination were incubated overnight at 4 °C. The following day, size exclusion chromatography was carried out on a HiLoad 16/60, Superdex G3000 column (GE Healthcare) equilibrated with 50 mM NaCl, 25 mM Tris, 5% (v/v) glycerol using an Agilent 1100 Series column (GE Healthcare).

MST. MicroScale Thermophoresis (MST) relates to the movement of particles subjected to a temperature gradient. When a temperature gradient is applied, changes in the hydration shell of proteins reflect protein structure/conformation changes, and this can be used to determine receptor:ligand binding affinities^43^. Both IL-6 and gp80 were labelled with NT647 using the Monolith labelling Red NHS kit (NanoTemper Technologies GmbH, Germany). NT647 dye is optimized for MST and carries a reactive NHS-ester group that modifies primary amines in lysine residue. Labelling and purification was carried out according to the protocol provided by the supplier. Standard treated capillaries NT.115 Series (NanoTemper Technologies GmbH, Germany) were used throughout the experiments. The red channel of Monolith NT.115 Blue/Red Monolith NT.115 (NanoTemper Technologies GmbH, Germany) was used. Both IL-6 and gp80 were labelled with NT647 dye with a ratio protein to dye above 0.5 to check for binding of IL-6 or gp80 only; a titration experiment using up to 20 μM VHH6 was performed. To determine the binding between VHH6 and IL-6–gp80, 10 nM IL-6NT647 and 20 nM gp80 were used, and VHH6 was titrated in, at a concentration starting with at least ten-fold the concentration of labelled molecules.

Octet. Octet is based on Bio-Layer Interferometry (BLI) for measuring e.g. protein:protein interactions and is a label-free technique. BLI analyzes the interference pattern of white light reflected from two surfaces: a layer of immobilized protein on the biosensor tip and an internal reference layer. BLI was used as a label-free technology for protein:protein binding^44^. The binding between VHH6 immobilized on the biosensor tip surface using Biosensor NiNTA tips (ForteBio, Pall Corporation) and FusionIL-6, IL-6, gp80 and IL-6–gp80 in complex was measured. Interference patterns for the binding to or dissociating from the biosensor were measured in real time to generate a response profile on the Octet^®^ System Octet 384 Red (ForteBio, Pall Corporation).

SPR measurements. All SPR experiments were carried out at 25 °C in HBS-P+ buffer (10 mM HEPES pH 7.4, 0.15 M NaCl, 0.05% (v/v) surfactant P20, GE Healthcare) as the running buffer. VHH6, IL-6, gp80 and gp130 proteins were immobilized on CM5 sensor chips (GE Healthcare) by standard amine coupling method, as recommended by the manufacturer. For continuity with X-ray crystallography and HDX-MS, gp80V4 was used in SPR measurements.

Qualitative SPR analysis was performed using a BIAcore 3000 (GE Healthcare); gp80 and VHH6 were injected at 50 nM concentration at a flow rate of 30 μl/min. gp80 was injected for 180 s and the dissociation was monitored for 360 s. Alternatively, gp80 was injected for 180 s and subsequently VHH6 was co-injected, and the dissociation was monitored for 360 s.

Quantitative SPR analysis was carried out using a BIAcore T200 (GE Healthcare). The binding measurements were performed at a flow rate of 100 μl/min. The protein of interest was injected for 180 s and the dissociation was monitored for 360 s. Binding of IL-6, gp80 and VHH6 was individually tested in a concentration series (0–250 nM, as two-fold serial dilution). When proteins were tested in combination, i.e. IL-6 and gp80, IL-6 and VHH6, gp80 and VHH6, gp80 and IL-6 and VHH6, one of the proteins was titrated in a concentration series (0–250 nM), while the other was kept constant at an excess concentration of 2 μM to ensure complex formation. The sensor surface was regenerated with 4 mM MgCl_2_. Background subtraction binding curves were analyzed using the T200 evaluation software (version 1.0) following standard procedures. Due to the complexity of the interactions studied, we have limited the data analysis to the most informative one, in this case the *k*_d_ parameter, which was determined by fitting a single exponential decay model to the dissociation data within 360 s range.

In order to evaluate the effect of VHH6 on the binding of the IL-6–gp80 complex to gp130, a 1:1 binding model was fitted to the kinetic data. The affinities for the interactions that reached equilibrium during the injection time of the experiment were determined from a steady state affinity fit, and are reported in the SI Tables. The binding curves were re-plotted in Prism (GraphPad Software Inc.) to render the figures with a better resolution.

Mutant versions of VHH6 containing single alanine substitutions at positions 27, 32, 74, 101 and 113, and double alanine substitutions at positions 27/32, 74/101 and 113/101 were generated, and supernatants produced in transient transfected cells (see paragraph Generation of VHH6 mutants) were tested by SPR. SPR analysis was performed using a BIAcore 3000 (GE Healthcare). His-tagged VHH6 and VHH6 mutants (1:5 diluted supernatants) were captured on a NTA chip (30 μl at 10 μl/min) following charging of the surface by injection of 10 μl 0.5 M NiCl_2_. An equimolar mixture of IL-6 and gp80 (50 nM each) was injected for 300 s at a flow rate of 10 μl/min, followed by a dissociation phase of 300 s. At the end of each cycle, the chip surface was regenerated by injection of 350 mM EDTA (2 × 10 μl).

*Cell culture*. Pooled HUVECs were purchased from Gibco (Thermo Fisher Scientific) and grown in 200PRF medium supplemented with Low Serum Growth Supplement Media (Gibco, Thermo Fisher Scientific). Different batches of HUVECs were tested in our experiments in order to have a good population representation. Cells were not used beyond the 5–6^th^ passage.

*p-STAT3 staining*. Cells were plated the day before at 1 × 10^4^ cells/well in 96well BD Falcon Becton Black/Clear tissue culture treated plates (Becton Dickinson). The following day, the cells were treated with FusionIL-6 (3 ng/ml), and IL-6+gp80 (respectively 10 ng/ml and 20 ng/ml) alone or in combination with an excess of VHH6, and fixed with PFA 4% at chosen time points (30 min, 180 min and 360 min). After 20 min incubation at RT, the cells were washed twice with PBS, and ice-cold methanol was added and stored at –20 °C. Before staining, the cells were washed twice in PBS, incubated for 20 min at 4 °C with PBS/10%FCS. Afterwards, anti-p-STAT3 (Tyr705) (clone D3A7, Cell Signalling) was added at a dilution suggested by the manufacturer (1:100 in PBS/10%FCS) and incubated for 1 h at 4 °C. After washing twice in PBS and secondary antibody goat anti-rabbit-AlexaFluor568 (Gibco, Thermo Fisher Scientific) was added and incubated for 1 h at 4 °C. Then, cells were washed twice in PBS, and the nuclei were stained for 10 min with a solution of DAPI in PBS/10%FCS (final concentration 2 μg/ml). Finally, cells were washed twice with PBS and 200 μl of fresh PBS were added to each well and stored in the dark at 4 °C.

*Internalization of the IL-6–gp80 complex*. Cells were plated the day before at 1 × 10^4^ cells/well in 96well BD BIOCOAT Cell Environment Collagen Cellware Black/Clear plates (Becton Dickinson). The following day the cells were treated with IL-6-NT647 alone (100 ng/ml), IL-6-NT647+gp80 (respectively 100 ng/ml and 200 ng/ml) with and without the addition of an excess of VHH6 and fixed with PFA 4% at chosen time points. After 20 min incubation at RT, the cells were washed twice with PBS, and ice-cold methanol was added and stored at −20 °C. Before staining, the cells were washed twice in PBS and incubated for 20 min at 4 °C with PBS/10%FCS. Afterwards, mouse anti-LAMP1 antibody (clone H4A3, Abcam) was added at a dilution suggested by the manufacturer (1:100 in PBS/10%FCS) and incubated for 1 h at 4 °C. After washing twice in PBS, secondary antibody goat anti-mouse-AlexaFluor488 (Gibco, Thermo Fisher Scientific) was added and incubated for 1 h at 4 °C. Then, cells were washed twice in PBS, and the nuclei were stained for 10 min with a solution of DAPI in PBS/10%FCS (final concentration 2 μg/ml). Finally, cells were washed twice with PBS, and 200 μl of fresh PBS were added to each well and stored in the dark at 4 °C.

*Image acquisition and analysis*. Images were acquired using Cellomics Arrayscan (Thermoscientific). For each well, 16-fields were captured containing approximately 100 cells per field at a resolution of 1104 × 1104 pixels. DAPI, Alexa488, Alexa568 and Alexa647 were detected using appropriate excitation and emission filter sets. Exposure times were kept constant between repeated experiments.

Cells were analyzed using the Cellomics Spot Detector Algorithm. Nuclei were detected on the basis of DAPI staining and STAT3 intensity was measured within this region. The “spot total intensity per object” parameter was used for further analysis.

For analysis of internalized IL-6-NT647, the mask for analysis was extended from the nuclei, and NT-647 intensity was measured from spots detected in this region.

*Statistical analysis*. The raw data from the Arrayscan analysis were processed using the “spot total intensity per object” parameter, and this parameter from three replicates was statistically analyzed using a linear mixed model for repeated measurements using the software package SAS 9.2 (SAS Institute, Cary NC).

*qPCR array*. Briefly, HUVECs were seeded at a concentration of 5 × 10^5^ cells/well in 6-well plates (Falcon, BD Biosciences) the day before. The following morning, the cells were treated with IL-6+gp80 and VHH6+IL-6+gp80 at the same concentration used for the pSTAT3 staining for 30, 180 and 360 min. At different time points, cells were washed twice with PBS, re-suspended in lysis buffer (Qiagen), immediately frozen in dry ice and stored at −80 °C.

Total RNA was extracted from HUVEC lysates using the RNeasy Plus Mini Kit (Qiagen) and quantitated using a Nanodrop (Thermo Fisher). Using the RT^2^ First Strand cDNA Kit (SABiociences, Qiagen) 400 ng of mRNA was converted to cDNA according to the manufacturer’s protocol. The Human IL-6/STAT3 Signalling Pathway Plus (PAHS-160YE-4) array, designed to quantitatively measure mRNA levels of 84 key genes involved IL-6 signaling in a 384-well format, was analyzed on an ABI 7900HT machine.

Data were analysed using the RT^2^ Profiler PCR array web based data analysis template v3.5 (http://pcrdataanalysis.sabiosciences.com/pcr/arrayanalysis.php) and changes in gene expression were calculated using the ΔΔC_t_ method with normalization of the raw data to housekeeping genes. Each RT^2^ Profiler PCR Array plates contained 84 genes related to the IL-6 pathway plus 5 different housekeeping genes. Three biological independent replicates of the same experiment were analyzed together. Only statistical significant results (p ≤ 0.05) were showed alongside the p-value.

*Structure determination*. X-ray crystallography The IL-6, gp80 and VHH6 complex was prepared using the engineered gp80V4 construct. The purified VHH6, gp80 and IL-6 were mixed in a molar ratio of 2:1:1 and incubated overnight at 4 °C to allow the complex formation. The complex was purified by size exclusion chromatography (SEC) over a HiLoad 16/60, Superdex 200 column (GE Healthcare) equilibrated with 50 mM NaCl, 25 mM Tris, 5% (v/v) glycerol. Fractions containing the complex were pooled and concentrated to 9 mg/ml for crystallography. Conditions suitable for crystal growth were identified by the sitting drop vapor diffusion method using commercially available crystallization screens (Qiagen). To generate diffraction quality crystals, hanging drop vapor diffusion method was used. 1 μl of protein solution was mixed with 1 μl of reservoir solution containing 0.1 M MES, pH 6.5, 14% PEG20K. Crystals reached full size in 14 d at 19 °C. Crystals were harvested and flash frozen in liquid nitrogen without additional cryo-protectant. Diffraction data to 2.7 Å were collected from a single crystal on the Proxima1 beamline at Soleil Synchrotron, France, and processed using XDS^46^. The structure of the complex was solved by molecular replacement with Phaser^47^, using coordinates of an in house VHH structure, and IL-6 and gp80 from PDB entry 1P9M^8^,^45^, as search models. The initial structure was refined with iterative cycles of simulated annealing, energy minimization and manual rebuilding using CNS^48^,^49^ and COOT^50^. Model geometry was validated using Molprobity^51^. Surface areas of the protein interfaces were calculated using PISA^52^. Molecular visualizations were generated with Pymol (DeLano W., The PyMOL Molecular Graphics System., 2002, version 1.7, DeLano Scientific LLC, San Carlos, CA. www.pymol.org). Data collection and refinement statistics are summarized in SI Table 2.

Generation of VHH6 mutants. To investigate the contribution to binding of IL-6 and gp80 by VHH6 residues identified in the crystal structure as being likely contact residues, we generated a panel of mutants using oligonucleotide-directed mutagenesis. Following small scale transient expression in Expi-HEK cells, supernatants were analyzed by SPR. The *k*d generated using supernatants from single and double mutants were compared to that of the supernatant from VHH6.

HDX-MS. 40 μM gp80V4–IL-6 or 40 μM gp80V4–IL-6–VHH6 complex, both purified by SEC, were diluted 20x either into 10 mM phosphate in H_2_O (pH 7.0), or into 10 mM phosphate in D_2_O (pD 7.0) and left to react for 0.5, 2, 10, 30 or 120 min. After reaction, these were diluted 1:1 with a cold quench solution (1 °C, 3.4 M Gdn.HCl, 500 mM TCEP, 200 mM phosphate) to give a final pH of 2.5. This mixture was immediately injected into the nanoAcquity HDX module (Waters Corp.) for peptic digest. All liquid handling steps were performed by a LEAP-PAL robot (CPC). The protein samples were washed onto the Enzymate pepsin column (25 °C) with 0.1% HCOOH in H_2_O at a flow-rate of 90 μL × min^−1^, and resulting peptides trapped on a VanGuard C18 trapping column over 3 min. These peptides were washed off the trap-column by reversing the flow and separated over a BEH C18 column (10 × 1.0 cm, 1.7 μm), using the following gradient: 0 min, 5% B; 7 min, 40% B; 8 min, 95% B; 10 min, 95% B, 11 min, 5% B (B: 0.2% HCOOH in H_2_O, A: 0.1% HCOOH in MeCN). Data acquisition was performed on a Synapt G2S (Waters Corp.) run in TOF-only mode over a m/z range of 50–2000 Th, using an MSe method (low collision energy, 10 V; high collision energy: ramp from 15 V to 35 V). Glu-1-Fibrinopeptide B peptide was used for internal lock mass correction.

HDX-MS Data Processing .Data were collected in triplicate, with blanks run between each data-point. MSe results from the H_2_O-diluted sample were analyzed using PLGS v3.0.1, searching for peptides against a database of the IL-6 and gp80V4 sequences only, with a precursor intensity threshold of 500 counts and 3 matched product ions required for assignment. Ion accounting files for the 6 control samples were combined into a peptide list imported into Dynamx v2.0 software, which was further curated to disallow peptides with a low energy intensity less than 100, peptides longer than 25 amino acids, and peptides eluting with a retention time longer than 9 min. Dynamx v2.0 was used to quantify the isotopic envelopes resulting from deuterium uptake for each peptide at each time-point; these data were then interrogated manually to ensure correct assignment of m/z peaks. Student’s t-tests were performed on the triplicate data-points for each time-point, and the significance of differential HDX between gp80V4–IL-6 and gp80V4–IL-6–VHH6 was defined as p < 0.01.

## Additional Information

**Accession codes**: Coordinates and structure factors were deposited in the PDB with the accession code 5FUC.

**How to cite this article**: Adams, R. *et al*. Discovery of a junctional epitope antibody that stabilizes IL-6 and gp80 protein:protein interaction and modulates its downstream signaling. *Sci. Rep.* **7**, 37716; doi: 10.1038/srep37716 (2017).

**Publisher's note:** Springer Nature remains neutral with regard to jurisdictional claims in published maps and institutional affiliations.

## Supplementary Material

Supplementary Information
