# Supplementary material for: Discovery of a junctional epitope antibody that stabilizes IL-6 and gp80 protein:protein interaction and modulates its downstream signaling
Source: Sci Rep. 2017 Jan 30;7:37716. doi: 10.1038/srep37716 (PMC5278397; doi:10.1038/srep37716)
Supplement: Supplementary Information [file srep37716-s1.pdf]

SUPPLEMENTARY INFORMATION (SI) FIGURES AND TABLES

Title: Discovery of a junctional epitope antibody that stabilizes IL-6 and gp80 protein:protein interaction and modulates its downstream signaling

Authors: Ralph Adams[1,#], Rebecca J. Burnley[1,#], Chiara R. Valenzano[1,#], Omar Qureshi[1], Carl Doyle[1], Simon Lumb[1], Maria del Carmen Lopez[1,^], Robert Griffin[1], David McMillan[1], Richard D. Taylor[1], Chris Meier[1], Prashant Mori[1], Laura M. Griffin[1], Ulrich Wernery[2], Jörg Kinne[2], Stephen Rapecki[1], Terry S. Baker[1], Alastair D. G. Lawson[1], Michael Wright[1], and Anna Ettorre[1,*]

Affiliations: [1]New Medicines, UCB-Celltech, 208 Bath Road, SL1 3WE, Slough UK; [2]Central Veterinary Research Laboratory, P.O.Box 597, Dubai, United Arab Emirates.

# These authors contributed equally to the work; * corresponding author.

SI Figure 1

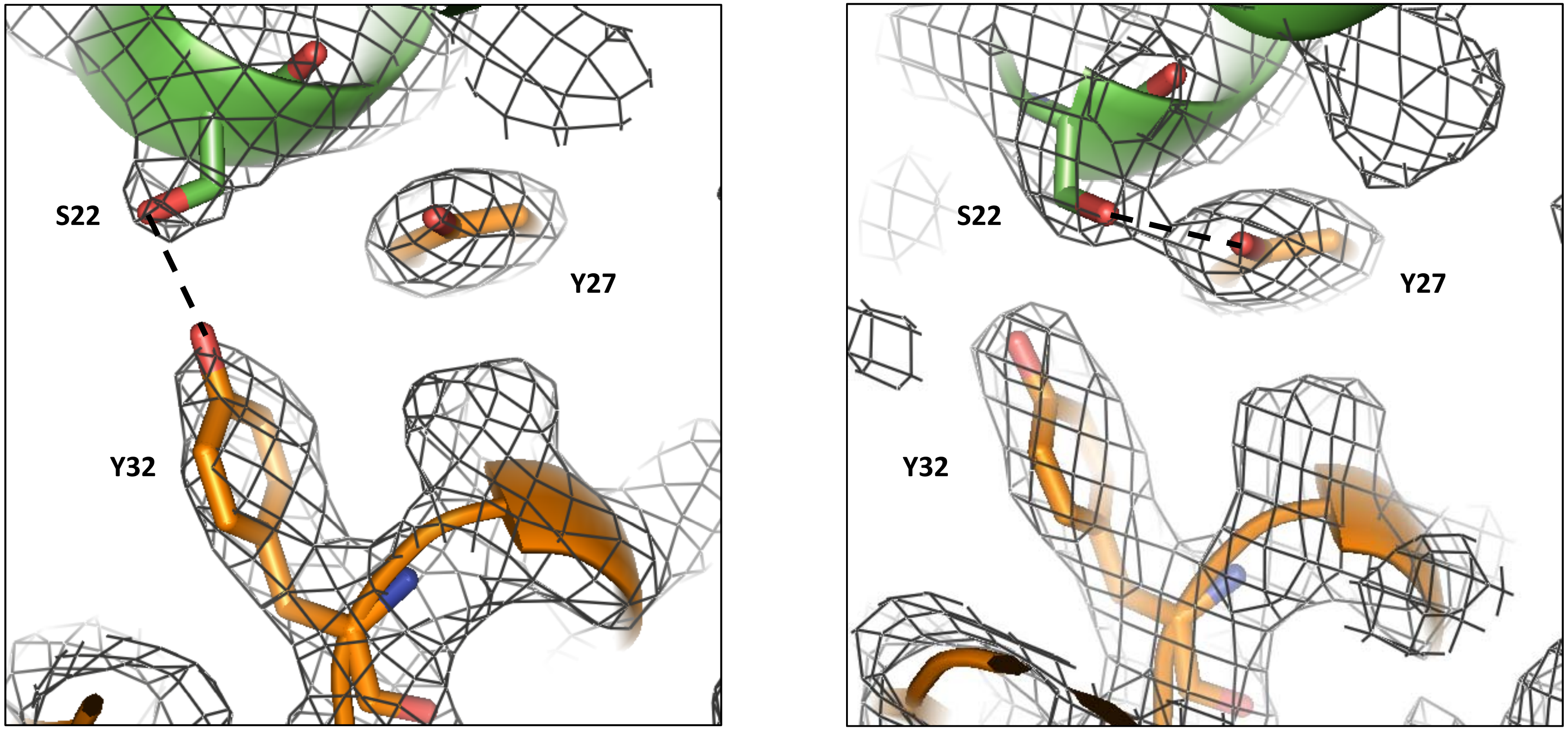

SI Figure 1. Differential hydrogen bonding in the two copies of the crystallographic asymmetric unit. There are two copies of the VHH6–IL-6–gp80 complex in the crystallographic asymmetric unit. In one copy, IL-6 (green) residue Ser22 forms a hydrogen bond with VHH6 (orange) residue Tyr32 (left), and in the second copy, Ser22 forms a hydrogen bond with VHH6 residue Tyr27 (right).

SI Figure 2

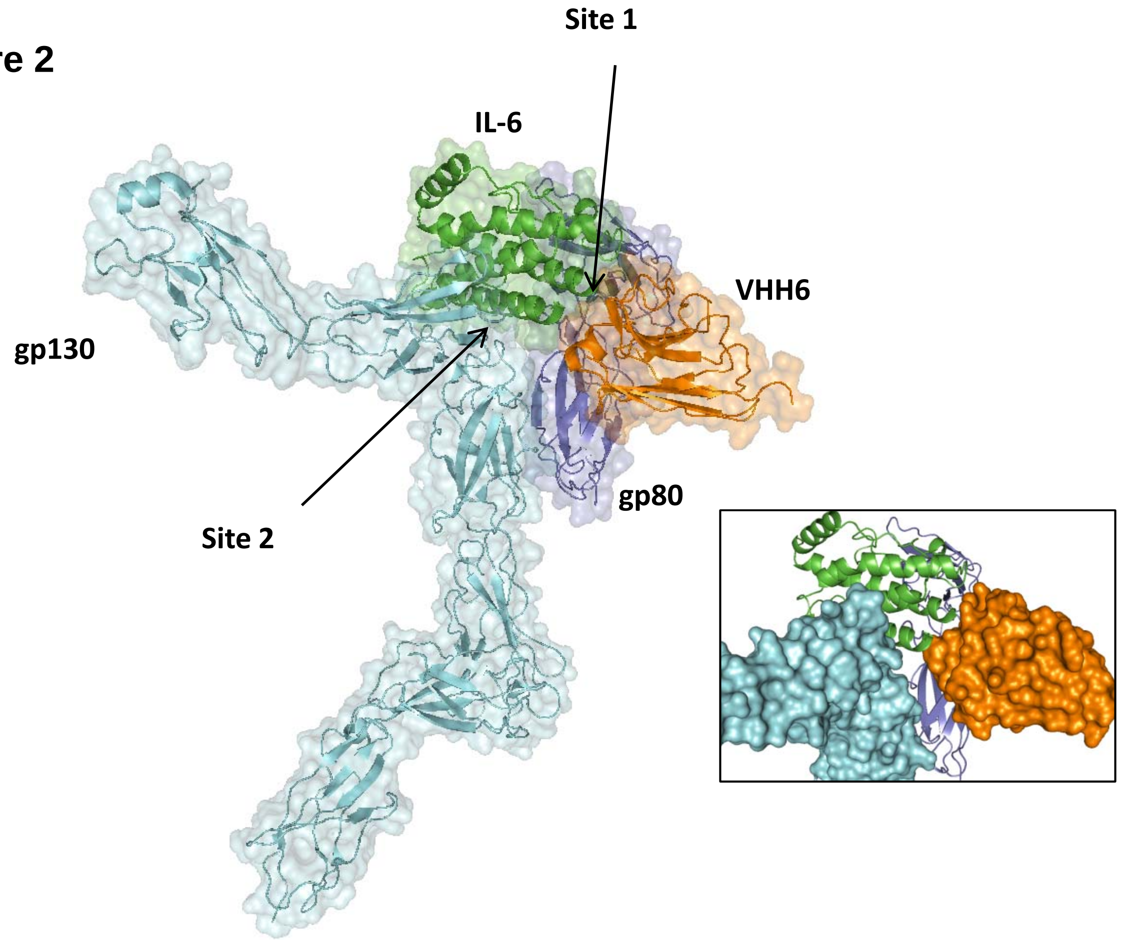

SI Figure 2. Model of crystal structure of VHH6–IL-6–gp80 superimposed with gp130.
Superimposition of IL-6 (green) and gp80 (blue) from the signaling complex, IL-6–gp80–gp130 (PDB code 1P9M), showed low r.m.s.d. values of 1.4±0.2 Å and 1.35±0.05 Å respectively, indicating that VHH6 (orange) holds IL-6 and gp80 together in a form able to bind gp130 (cyan)**.**

SI Figure 3

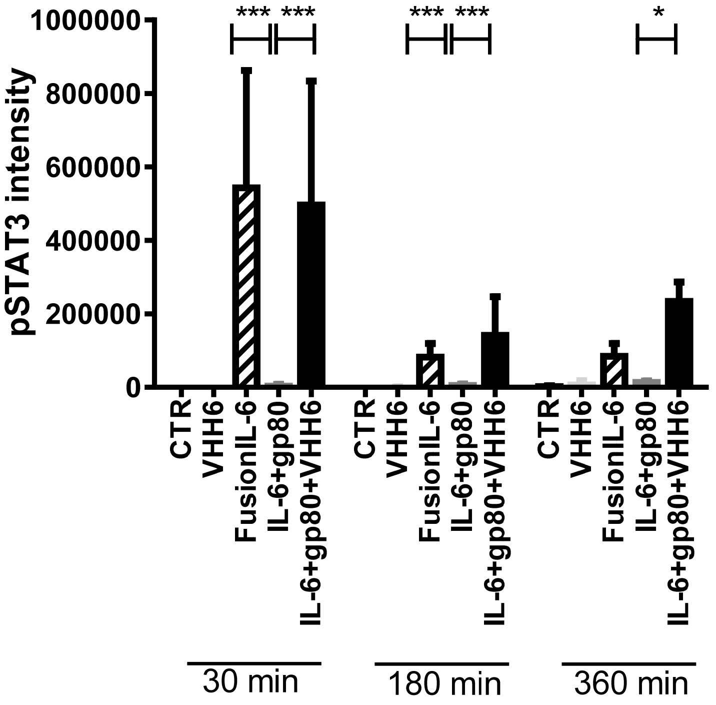

SI Figure 3. Stabilization of the IL-6–gp80 complex by VHH6 promotes higher and sustained STAT3 phosphorylation signal in HUVECs comparable to FusionIL-6 fusion protein.
HUVECs were treated with FusionIL-6, IL-6+gp80 and VHH6+IL-6+gp80 as described in Materials and Methods. STAT3 phosphorylation signal was quantified at different time points using the “spot total intensity per object” parameter as described in Materials and Methods. Statistical analysis of pSTAT3 signal from three replicates was performed for each time point (statistical significance: *=p≤0.05; **=p≤0.01 and ***=p≤0.001). Unlike IL-6+gp80, in the presence of VHH6 or when FusionIL-6 was added to the culture, pSTAT3 was increased at earlier (30 min) and later time points (180 min and 360 min). y-axis: pSTAT3 fluorescence expressed as spot total intensity per object; x-axis: time (min).

SI Figure 4

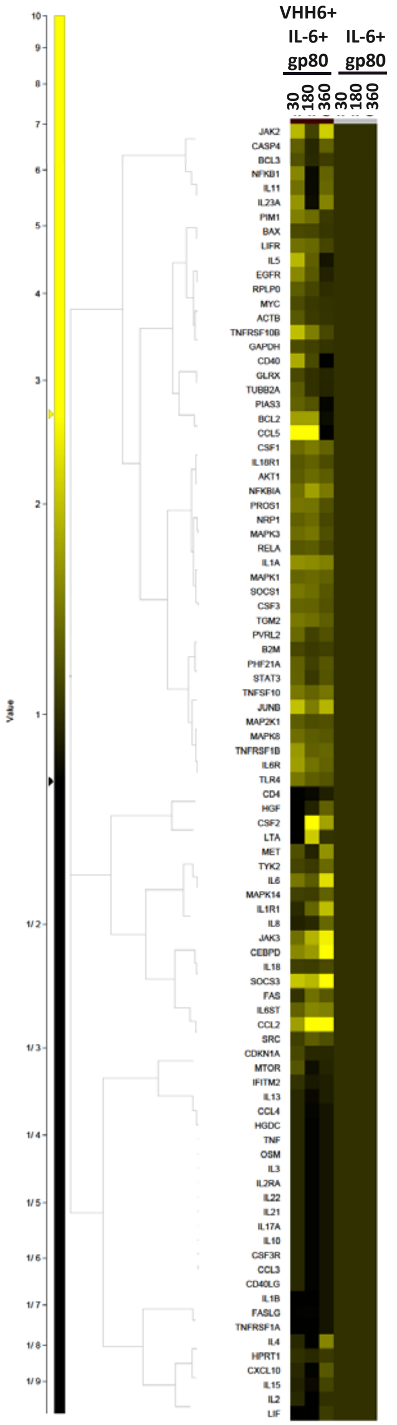

SI Figure 4. Transcriptomic analysis of HUVECs treated with VHH6+IL-6+gp80 confirms selective up-regulation of pro-inflammatory genes.
Three different batches of HUVECs were analyzed at 30, 180 and 360 min. VHH6+IL-6+gp80 (sample) were compared to IL-6+gp80 (control). Data were analyzed using the RT^2 Profiler PCR array web based data analysis template v3.5 (http://pcrdataanalysis.sabiosciences.com/pcr/arrayanalysis) and changes in gene expression changes were calculated using the $\Delta\Delta C_t$ method with normalization of the raw data to housekeeping genes. A heat map was generated using Genedata's Analyst software. Genes in grey-black are down-regulated, while genes in yellow are up-regulated. Gene regulation for each sample is expressed as fold change compared to control.

SI Table 1

Table 1A: Data collection and refinement statistics (molecular replacement)

	**Junctional Epitope Antibody in complex with IL-6 and gp80**
Data collection	
**Space group**	C 1 2 1
**Cell dimensions**	
a, b, c **(Å)**	249.03, 67.80, 78.16
α, β, γ **(°)**	90.00, 104.53, 90.00
**Resolution (Å)**	47.84-2.70(2.86-2.69)*
R_{meas} **(%)**	7.2(50.1)
$CC_{1/2}$ **(%)**	99.9(91.5)
$I / \sigma I$	20.88(3.97)
**Completeness (%)**	99.4(96.9)
**Redundancy**	7.5(7.5)
Refinement	
**Resolution (Å)**	20.00-2.70
**No. reflections**	34,870
R_{work}/R_{free}	0.2313/0.2872
**No. atoms**	
**Protein**	7590
**Water**	111
***B*-factors**	
**Protein**	56.33
**Water**	43.19
**R.m.s. deviations**	
**Bond lengths (Å)**	0.007
**Bond angles (°)**	1.289

*Values in parentheses are for highest-resolution shell.

SI Table 1B: Single mutants of VHH6

	***k*d (1/s)**
wtVHH6	3.6E-04
N74A	1.5E-04
K113A	2.6E-04
Y32A	3.0E-04
S101A	3.9E-04
Y27A	7.0E-04

SI Table 1C: Double mutants of VHH6

	***k*d (1/s)**
wtVHH6	3.6E-04
Y27A Y32A	4.3E-04
N74A S101A	13.5E-04
K113A S101A	14.3E-04
K113S S101A	16.4E-04

SI Table 1. Additional data supporting the junctional epitope nature of VHH6.

(A) Data collection and refinement statistics (molecular replacement). To assess the contributions of the side chains to binding, alanine-scanning was carried out. The dissociation rate of single **(B)** and double mutants **(C)** were assessed using SPR.

SI Table 2: Peptides identified and quantified in IL-6 and gp80 by HDX-MS

SI Table 2A: Peptide identified in IL-6

Start Residue	End Residue	Sequence	2 timepoints with p<0.01	4 timepoints with p<0.01
**0**	11	APVPPGEDSKDV		
**3**	23	PPGEDSKDVAAPHRQPLTSSE		
**21**	28	SSERIDKQ	*	
**21**	31	SSERIDKQIRY	*	
**22**	31	SERIDKQIRY	*	
**24**	33	RIDKQIRYIL	*	*
**25**	36	IDKQIRYILDGI	*	*
**29**	39	IRYILDGISAL		
**38**	49	ALRKETCNKSNM	#	
**39**	49	LRKETCNKSNM	#	
**40**	49	RKETCNKSNM	#	
**40**	51	RKETCNKSNMCE		
**41**	61	KETCNKSNMCESSKEALAENN	#	
**42**	56	ETCNKSNMCESSKEA		
**52**	75	SSKEALAENNLNLPKMAEKDGCFQ		
**53**	63	SKEALAENNLN		
**53**	75	SKEALAENNLNLPKMAEKDGCFQ		
**55**	65	EALAENNLNLP		
**58**	67	AENNLNLPKM		
**58**	74	AENNLNLPKMAEKDGCF	*	*
**59**	80	ENNLNLPKMAEKDGCFQSGFNE	*	
**60**	67	NNLNLPKM		
**60**	70	NNLNLPKMAEK		
**60**	82	NNLNLPKMAEKDGCFQSGFNEET	*	
**68**	74	AEKDGCF	*	*
**70**	81	KDGCFQSGFNEE	*	
**75**	84	QSGFNEETCL		
**85**	92	VKIITGLL		
**85**	93	VKIITGLLE		
**87**	93	IITGLLE		
**98**	108	LEYLQNRFESS		
**99**	109	EYLQNRFESSE		
**99**	110	EYLQNRFESSEE		
**99**	112	EYLQNRFESSEEQA		

**99**	120	EYLQNRFESSEEQARAVQMSTK		
**100**	110	YLQNRFESSEE		
**100**	112	YLQNRFESSEEQA		
**104**	114	RFESSEEQARA		
**106**	112	ESSEEQA		
**106**	116	ESSEEQARAVQ		
**109**	116	EEQARAVQ		
**115**	122	VQMSTKVL		
**115**	122	VQMSTKVL		
**120**	126	KVLIQFL		
**123**	135	IQFLQKKAKNLDA		
**126**	133	LQKKAKNL	*	
**126**	135	LQKKAKNLDA	*	
**126**	144	LQKKAKNLDAITTPDPTTN	*	
**126**	145	LQKKAKNLDAITTPDPTTNA		
**126**	147	LQKKAKNLDAITTPDPTTNASL	*	
**129**	143	KAKNLDAITTPDPTT		
**136**	147	ITTPDPTTNASL	*	*
**148**	155	LTKLQAQN		
**148**	155	LTKLQAQN		
**148**	158	LTKLQAQNQWL		
**148**	160	LTKLQAQNQWLQD		
**152**	158	QAQNQWL		
**159**	165	QDMTTHL	*	
**159**	167	QDMTTHLIL	*	
**161**	167	MTTHLIL	*	
**166**	172	ILRSFKE	*	*
**166**	173	ILRSFKEF	*	*
**166**	174	ILRSFKEFL	*	
**168**	174	RSFKEFL	*	*
**168**	178	RSFKEFLQSSL	*	*

SI Table 2B: SI Table 2A: Peptide identified in gp80

Start Residue	End Residue	Sequence	2 timepoints with p<0.01	4 timepoints with p<0.01
**66**	86	HENLYFQGLAPRRCPAQEVAR		
**73**	82	GLAPRRCPAQ		
**73**	83	GLAPRRCPAQE	*	
**73**	85	GLAPRRCPAQEVA	*	
**78**	96	RCPAQEVARGAGAGDVPPE		
**81**	102	AQEVARGAGAGDVPPEEPQLSC	*	
**82**	95	QEVARGAGAGDVPP		
**83**	94	EVARGAGAGDVP	*	
**84**	100	VARGAGAGDVPPEEPQL	*	
**86**	100	RGAGAGDVPPEEPQL	*	
**92**	110	DVPPEEPQLSCFRKSPLSN	*	
**94**	100	PPEEPQL		
**103**	111	FRKSPLSNV		
**105**	123	KSPLSNVVCEWGPRSTPSL		
**111**	123	VVCEWGPRSTPSL		
**112**	123	VCEWGPRSTPSL		
**115**	129	WGPRSTPSLTTKAVL		
**124**	130	TTKAVLL		
**130**	141	LVRKFQNSPAED		
**131**	141	VRKFQNSPAED		
**135**	145	QNSPAEDFQEP		
**139**	158	AEDFQEPCQYSQESQKFSCQ		
**141**	147	DFQEPCQ		
**146**	164	CQYSQESQKFSCQLAVPEG		
**147**	164	QYSQESQKFSCQLAVPEG		
**148**	158	YSQESQKFSCQ		
**152**	158	SQKFSCQ		
**157**	170	CQLAVPEGDSSFYI		
**159**	168	LAVPEGDSSF	*	
**162**	168	PEGDSSF	*	
**174**	183	CVASSVGSKF		
**176**	183	ASSVGSKF		
**195**	205	LQPDPPANITV	*	
**201**	210	ANITVTAVAR		
**206**	216	TAVARNPRWLS	*	
**207**	214	AVARNPRW		
**208**	214	VARNPRW		
**208**	217	VARNPRWLSV	*	

**208**	218	VARNPRWLSVT	*	
**218**	229	TWQDPHSWNSSF	*	
**221**	229	DPHSWNSSF		
**230**	236	YRLRFEL		
**235**	245	ELRYRAERSKT		
**237**	245	RYRAERSKT		
**243**	256	SKTFTTWMVKDLQH	*	*
**250**	262	MVKDLQHHAVIHD	*	
**250**	266	MVKDLQHHAVIHDAWSG	*	*
**250**	273	MVKDLQHHAVIHDAWSGLRHVVQL	*	
**265**	273	SGLRHVVQL		
**267**	273	LRHVVQL		
**268**	275	RHVVQLRA		
**274**	283	RAQEEFGQGE	*	*
**274**	284	RAQEEFGQGEW	*	*
**274**	286	RAQEEFGQGEWSE	*	*
**277**	295	EEFGQGEWSEWSPEAMGTP		
**280**	296	GQGEWSEWSPEAMGTPW		
**284**	291	WSEWSPEA	*	
**284**	298	WSEWSPEAMGTPWTE		
**284**	303	WSEWSPEAMGTPWTESRSPP		
**285**	291	SEWSPEA		
**285**	298	SEWSPEAMGTPWTE		
**285**	303	SEWSPEAMGTPWTESRSPP		
**287**	298	WSPEAMGTPWTE		
**289**	303	PEAMGTPWTESRSPP		
**292**	298	MGTPWTE		

SI Table 2: Additional data supporting HDX-MS.

List of peptides identified in IL-6 **(A)** and gp80 **(B)** and quantified by HDX-MS. Asterisk (*) indicates deuterium uptake increasing the presence of VHH6; hash (#) indicates deuterium uptake decreasing in the presence of VHH6.

SI Table 3: SPR analysis

IL-6 immobilized	*k*d (1/s)	SE (*k*d)	KD (M)	SE (KD)
**gp80**	0.046	5.40E-05	3.42E-08	7.20E-09
**gp80+VHH6 (2 μM)**	1.94E-04	1.90E-07	NA	

gp80 immobilized	*k*d (1/s)	SE (*k*d)	KD (M)	SE (KD)
**IL-6**	0.048	4.70E-05	1.54E-08	1.50E-09
**IL-6+VHH6 (2 μM)**	2.10E-04	2.80E-07	NA	

VHH6 immobilized	*k*d (1/s)	SE (*k*d)	KD (M)
**IL-6+gp80 (2 μM)**	3.09E-04	4.40E-07	NA
**gp80+IL-6 (2 μM)**	3.75E-04	6.00E-07	NA

gp130 immobilized	*k*a (1/Ms)	*k*d (1/s)
**gp80 [IL-6 (2 μM)]**	3.6 10^5	0.037
**gp80 [IL-6 (2 μM)+VHH6 (2 μM)]**	4.7 10^5	0.029
**IL-6 [gp80 (2 μM)]**	3.6 10^5	0.035
**IL-6 [gp80 (2 μM) + VHH6 (2 μM)]**	4.4 10^5	0.036

SI Table 3: Additional SPR data analysis.

During SPR studies binding of IL-6, gp80 and VHH6 were individually tested in a concentration series (0-250 nM, as two-fold serial dilution). When proteins were tested in combinations (IL-6 and gp80, IL-6 and VHH6, gp80 and VHH6, gp80 and IL-6 and VHH6) one of the proteins was titrated in a concentration series (0-250 nM), while the other was kept constant at an excess concentration of 2 µM (in brackets) to ensure complex formation. A total of four proteins were immobilized on the chip, from top to bottom: IL-6, gp80, VHH6 and gp130.

SI Table 4: In-house constructs

Construct	Expression Host	Promoter	Affinity Tag	Expressed Protein
**pNAFL-8His-fusionIL-6(gp80D123)**	CHO	CMV	8His	gp80(L20-S320)(C211A, C277A)-IL-6(V30-M212)
**pNAFL-gp80D123-hscFc**	CHO	CMV	Single chain human IgG Fc fragment	gp80(M1-P322)(C211A, C277A)-hscFc
**pNAFL-8His-gp80V4**	CHO	CMV	8His	gp80V4
**pTrx-6His-hIL-6**	*E.coli*	T7	Thioredoxin-6His	Trx-6His-IL-6(A28-M212)
**pTrx-6His-IL-6(S21)**	*E.coli*	T7	Thioredoxin-6His	Trx-6His-IL-6(S49-M212)
**pIMMs-6His-VHH6**	Expi-HEK	CMV	6His	VHH6-6His

SI Table 4: In-house designed constructs.

All constructs listed in the table were designed in-house to support the immunization campaign, antibody screening, junctional antibody characterization and biophysics analysis.
